# Body Composition in Patients with Radioactive Iodine-Refractory, Advanced Differentiated Thyroid Cancer Treated with Sorafenib or Placebo: A Retrospective Analysis of the Phase III DECISION Trial

**DOI:** 10.1089/thy.2018.0784

**Published:** 2019-12-16

**Authors:** Olivier Huillard, Anne Jouinot, Camille Tlemsani, Marcia S. Brose, Jennifer Arrondeau, Gerold Meinhardt, Marc Fellous, Yoriko De Sanctis, Martin Schlumberger, Francois Goldwasser

**Affiliations:** ^1^ Department of Medical Oncology, Cochin Hospital, AP-HP, Paris, France.; ^2^ Department of Medical Oncology, Paris Descartes University, CARPEM, Paris, France.; ^3^ Department of Otorhinolaryngology, Head and Neck Surgery, Abramson Cancer Center, University of Pennsylvania, Perelman School of Medicine, Philadelphia, Pennsylvania.; ^4^ Clinical Development Oncology; Bayer HealthCare Pharmaceuticals, Whippany, New Jersey.; ^5^ Pharmaceuticals Division; Bayer HealthCare Pharmaceuticals, Whippany, New Jersey.; ^6^ Integrated Analysis Statistics, Bayer HealthCare Pharmaceuticals, Whippany, New Jersey.; ^7^ Nuclear Medicine and Endocrine Oncology, Institut Gustave Roussy, Villejuif, France.

**Keywords:** differentiated thyroid carcinoma, sorafenib, toxicity, dose modification, sarcopenia

## Abstract

***Background:*** Rates of adverse events with sorafenib were higher in the DECISION trial in radioactive iodine-refractory, advanced differentiated thyroid cancer (DTC) than in trials of sorafenib for other tumor types. One possible explanation is that sarcopenia, a known predictive factor of toxicity in patients with cancer, is more common in patients with DTC due to hormone suppressive therapy.

***Methods:*** This retrospective exploratory analysis was performed to assess whether the risk of early toxicity leading to dose modification (DMT) with sorafenib was higher in patients with sarcopenia compared with those without sarcopenia. The data set comprised patients from the phase III DECISION trial with a computed tomography scan available to determine muscle mass. The skeletal muscle (SM) cross-sectional area was used to determine the SM index and define sarcopenia. The end points were changes in body composition, DMT, early DMT (within 1 month), severe toxic events (STEs), and early STEs.

***Results:*** Overall, 365 patients were eligible for this analysis; baseline characteristics were well balanced between patients receiving sorafenib (*n* = 180) versus placebo (*n* = 185). Using a sarcopenia definition of an SM index less than the median sex-specific SM index, approximately half of the patients receiving sorafenib were at risk of sarcopenia (89/180; 49.4%), with wide geographical variation. At 6 months, the mean weight, body mass index, and lean body mass of patients receiving sorafenib were lower than at baseline and significantly lower than for patients receiving placebo (all *p* < 0.0001). Most DMTs and STEs occurred in the first month of treatment. There was a nonsignificant trend for more early DMTs in patients with sarcopenia compared with those without sarcopenia (55.3% vs. 44.7%, respectively; *p* = 0.2273).

***Conclusions:*** These results show a significant effect of sorafenib on muscle mass. However, there was no association between sarcopenia and DMT or early DMT, in contrast to observations in hepatocellular and renal cell carcinoma.

## Introduction

Sarcopenia, characterized by loss of skeletal muscle (SM) mass, is an adverse prognostic factor in many types of cancer (1–4); however, little is known about its pathophysiology in patients with cancer (2). A recent meta-analysis showed that a low SM index at cancer diagnosis is associated with worse survival in patients with solid tumors (5). Sarcopenia is an independent prognostic factor for complications and survival after cancer surgery and is associated with poor performance status, toxicity from chemotherapy, and shorter duration of tumor control in patients with advanced cancer (4,6,7). Sarcopenia may also predict the toxicity of targeted agents, such as tyrosine kinase inhibitors.

Sorafenib is a multikinase inhibitor (8,9) approved for the treatment of patients with advanced hepatocellular carcinoma (HCC), advanced renal cell carcinoma (RCC), and progressive, locally advanced, or metastatic differentiated thyroid carcinoma (DTC) refractory to radioactive iodine. In patients with HCC, sarcopenia has been associated with sorafenib toxicity (10,11), and sarcopenia was a significant predictor of sorafenib and sunitinib toxicity in patients with RCC with a body mass index (BMI) of <25 kg/m^2^ (11,12). In HCC, the higher incidence of adverse events (AEs) in sarcopenic patients was linked to higher sorafenib exposure (13). This may also explain the link between sarcopenia and poor clinical outcomes in sorafenib-treated patients with HCC (14), consistent with the observation that muscle wasting is an important prognostic factor in patients treated with sorafenib (15).

The approval of sorafenib for DTC was based on results of the phase III DECISION trial (16), in which sorafenib improved the primary end point of progression-free survival (PFS) compared with placebo (median PFS: 10.8 vs. 5.8 months, respectively; *p* < 0.0001) (16). Although AEs with sorafenib were typically grade 1–2 and mostly occurred early in the treatment course (17), rates of dose modifications (DMTs) with sorafenib were higher than in phase III trials of sorafenib in RCC and HCC (18,19). One possible explanation for the increase in toxicity leading to DMT with sorafenib in the DECISION trial could be a greater prevalence of sarcopenia following long-term thyroxine suppressive therapy (20). To investigate this, we conducted a subgroup analysis of the DECISION trial to determine whether sarcopenic patients with DTC have a higher risk of DMTs during the first month of sorafenib treatment than those without sarcopenia.

## Materials and Methods

### Study population

DECISION was a multicenter, randomized, double-blind, placebo-controlled phase III trial (NCT00984282; EudraCT 2009-012007-25) evaluating sorafenib (400 mg twice daily) versus placebo in patients with locally advanced or metastatic radioactive iodine-refractory DTC. Details of the DECISION trial have been previously published (16).

The analysis data set comprised patients in the DECISION trial with a lumbar computed tomography (CT) scan taken ≥30 days before the start of treatment that could be analyzed to determine muscle mass. The reasons for exclusion from the analysis set were a lack of an appropriate lumbar CT scan or the presence of prosthetic metal that prevented analysis of the scan.

### Assessments

Skeletal muscle mass and lean body mass (LBM) were determined using methods previously described (10–13,21). CT images taken for diagnostic and follow-up purposes ≥30 days before the initiation of sorafenib were used to assess regional muscle mass. Images were analyzed using ImageJ software, v1.42q (National Institutes of Health). The third lumbar vertebra (L3) was used as a standard landmark; the cross-sectional areas (cm^2^) of the sum of all muscles were computed for each image, and the mean values for two consecutive images were computed for each patient. The total lumbar SM cross-sectional area (SM area; cm^2^) was normalized for stature and expressed in cm^2^/m^2^. The SM index (cm^2^/m^2^) was calculated using the formula: SM index (cm^2^/m^2^) = SM area (cm^2^)/(0.01 × height)^2^ (m^2^). Total LBM was estimated from the muscle cross-sectional areas using the formula: LBM (kg) = (0.30 × (SM area (cm^2^))+6.06). Three different sets of criteria (A–C) were used to define sarcopenia:

 ○ Definition A [based on Ref. (22)]:
 ○ Men: SM index <55.4 cm^2^/m^2^
 ○ Women: SM index <38.9 cm^2^/m^2^
 ○ Definition B [based on Refs. (10,11)]:
 ○ Men: SM index <55.4 cm^2^/m^2^ and BMI <25 kg/m^2^
 ○ Women: SM index <38.9 cm^2^/m^2^ and BMI <25 kg/m^2^
 ○ Definition C:
 ○ Men: SM index less than the median SM index value for men in the sarcopenia analysis set (SAS)
 ○ Women: SM index less than the median SM index value for women in the SAS

The end points assessed were changes in mean weight, BMI, and LBM from baseline to 6 months; DMTs within 6 months from the initiation of therapy, defined as any AE leading to DMT (reduction, interruption, or permanent discontinuation); early DMTs (DMTs in the first 30 days of treatment); severe toxic events (STEs) within 6 months, defined as any grade 3–5 toxic event; and early STEs (STEs occurring within the first 30 days of treatment).

### Statistical analyses

Descriptive statistics were applied for the baseline characteristics of the SAS. Mean changes from baseline to 6 months in weight, BMI, and LBM were compared between sorafenib and placebo groups using Student's *t*-test (normal distribution) or the Wilcoxon rank-sum test and with the paired *t*-test (normal distribution) or Wilcoxon matched-pairs signed-rank test. Time to DMT and time to STE were analyzed for patients with and without sarcopenia. Patients who were no longer in the study at 6 months or who did not have 6-month scans were censored at 6 months for the time-to-event analysis and treated as missing for other analyses.

Univariate analysis was performed based on logistic regression models with the dependent variables of early DMT and baseline characteristics of interest. Categorical variables were compared between groups (early DMT or no early DMT) with Fisher's exact test. Continuous variables were compared between groups with Student's *t*-test. Appropriate transformation was applied for variables for which a normal distribution could not be assumed. Backward, stepwise multiple logistic regression was performed to investigate prognostic factors for early DMT using potential explanatory variables selected from the univariate analysis (*p* ≤ 0.20). In addition, factors considered to be clinically relevant or potential confounders were included in the model. A significance level of 5% was used to decide whether a factor should remain as a variable in the analysis. A multivariate analysis was undertaken using the Cox proportional hazard regression model to investigate time to STE. All statistical analyses were exploratory, and reported *p*-values should be interpreted with caution. No adjustments for multiplicity were made.

## Results

### Baseline characteristics

**FIG. 1.**
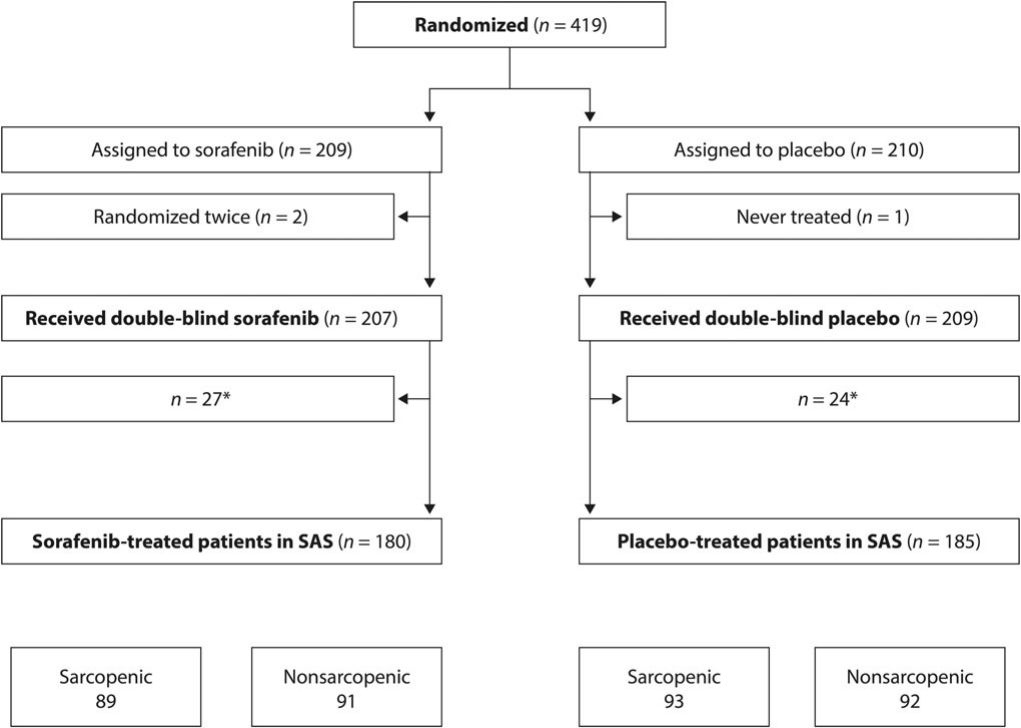
Patient disposition. *The reason for exclusion from the SAS was a lack of an appropriate lumbar CT scan or the presence of a prosthetic metal preventing analysis of the CT scan. CT, computed tomography; SAS, sarcopenia analysis set.

Of the 419 subjects randomized in the DECISION trial, 365 were eligible for inclusion in this analysis (sorafenib, *n* = 180; placebo, *n* = 185) (Fig. 1). Baseline characteristics were well balanced between the two groups (Table 1). As expected, mean weight, SM area, SM index, and LBM were lower in females than males. Given the heterogeneity of the population, definition C for sarcopenia was used for all analyses presented here. Approximately half of the sorafenib-treated patients were sarcopenic (89/180; 49.4%), with wide geographic variation: 46/104 (44.2%) European patients, 10/31 (32.3%) North American patients, and 33/45 (73.3%) Asian patients were sarcopenic at baseline. In addition, at baseline, patients with sarcopenia were older and had a lower mean body weight, BMI, SM area, SM index, and LBM than nonsarcopenic patients (Table 2). Baseline characteristics for patients with sarcopenia versus those without sarcopenia for all definitions (A–C) are shown in Supplementary Table S1.

**Table 1.** Baseline Characteristics and Body Composition

Variable	Placebo (*n* = 185)	Sorafenib (*n* = 180)
Age, years		
Median (range)	62 (30–87)	63 (24–82)
Mean (SD)	61.1 (11.8)	61.5 (11.3)
ECOG PS, *n* (%)		
0	116 (62.7)	113 (62.8)
1	62 (33.5)	60 (33.3)
2	6 (3.2)	6 (3.3)
Missing	1 (0.5)	1 (0.6)
Time since diagnosis, months		
Median (range)	69 (67 (4–363)	
Mean (SD)	93.4 (70.5)	83.8 (72.9)
Weight, kg		
Median (range)	73 (42–142)	75 (35–140)
Mean (SD)	75.6 (19.2)	75.6 (18.2)
BMI, kg/m^2^		
Median (range)	26 (16–48)	27 (16–45)
Mean (SD)	27.1 (5.9)	26.7 (5.1)
BMI group, *n* (%)		
<18.5 kg/m^2^	5 (2.7)	7 (3.9)
18.5–24.9 kg/m^2^	73 (39.5)	64 (35.6)
25–29.9 kg/m^2^	58 (31.4)	66 (36.7)
≥30 kg/m^2^	49 (26.5)	43 (23.9)
SM index, cm^2^/m^2^		
Median (range)	44 (25–81)	46 (26–75)
Mean (SD)	45.8 (9.9)	46.3 (9.2)
LBM, kg		
Median (range)	43 (25–79)	45 (27–74)
Mean (SD)	44.5 (10.5)	45.6 (10.6)
Region, *n* (%)		
Europe	108 (58.4)	104 (57.8)
North America	31 (16.8)	31 (17.2)
Asia	46 (24.9)	45 (25.0)

BMI, body mass index; ECOG PS, Eastern Cooperation Oncology Group performance status; LBM, lean body mass; SD, standard deviation; SM, skeletal muscle.

**Table 2.** Baseline Characteristics and Body Composition According to Sarcopenia Status (Definition C)

	Sarcopenic		Nonsarcopenic	
	Placebo (*n* = 93)	Sorafenib (*n* = 89)	Placebo (*n* = 92)	Sorafenib (*n* = 91)
Age, years				
Median (range)	65 (33–87)	65 (24–82)	59 (30–80)	62 (27–80)
Mean (SD)	63.5 (12.2)	63.0 (11.5)	58.7 (11.0)	60.1 (11.1)
ECOG PS, *n* (%)				
0	58 (62.4)	50 (56.2)	58 (63.0)	63 (69.2)
1	32 (34.4)	33 (37.1)	30 (32.6)	27 (29.7)
2	3 (3.2)	6 (6.7)	3 (3.3)	0
Missing	0	0	1 (1.1)	1 (1.1)
Time since diagnosis, months				
Median (range)	97 13–402)	66 (4–363)	54 (7–325)	67 (4–348)
Mean (SD)	109.6 (73.7)	86.5 (75.0)	76.7 (63.3)	81.0 (71.0)
Weight, kg				
Median (range)	66 (44–125)	66 (35–120)	82 (42–142)	83 (44–140)
Mean (SD)	68.5 (14.8)	68.5 (15.9)	82.7 (20.6)	82.6 (17.6)
BMI, kg/m^2^				
Median (range)	24 (16–39)	23 (16–35)	29 (17–48)	29 (18–45)
Mean (SD)	24.4 (4.0)	24.1 (3.8)	29.9 (6.2)	29.3 (4.9)
BMI group, *n* (%)				
<18.5 kg/m^2^	3 (3.2)	6 (6.7)	2 (2.2)	1 (1.1)
18.5–24.9 kg/m^2^	56 (60.2)	48 (53.9)	17 (18.5)	16 (17.6)
25–29.9 kg/m^2^	24 (25.8)	31 (34.8)	34 (37.0)	35 (38.5)
≥30 kg/m^2^	10 (10.8)	4 (4.5)	39 (42.4)	39 (42.9)
SM index, cm^2^/m^2^				
Median (range)	39 (25–51)	39 (26–51)	53 (40–81)	53 (40–75)
Mean (SD)	39.5 (6.1)	39.9 (6.2)	52.2 (8.9)	52.6 (7.2)
LBM, kg				
Median (range)	38 (25–58)	38 (27–58)	49 (32–79)	52 (34–74)
Mean (SD)	39.6 (7.8)	40.1 (7.9)	49.6 (10.4)	50.9 (10.2)

Sarcopenia definition C = men: SM index less than the median SM index value for men; women: SM index less than the median SM index value for women.

BMI, body mass index; ECOG PS, Eastern Cooperation Oncology Group performance status; LBM, lean body mass; SD, standard deviation; SM, skeletal muscle.

### Six-month body composition of patients receiving sorafenib versus placebo

**FIG. 2.**
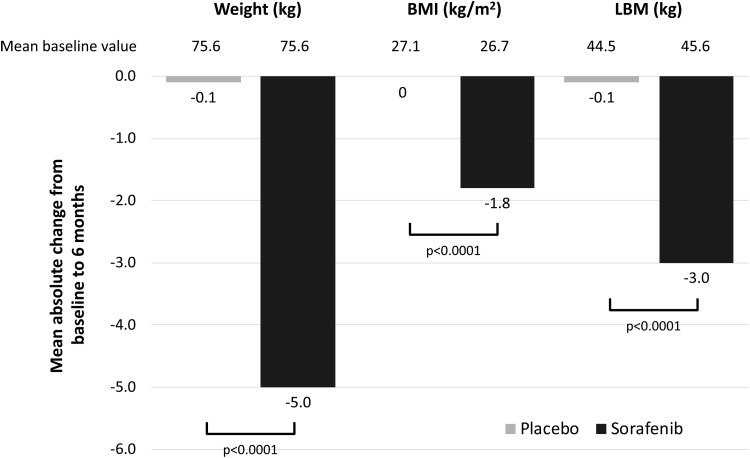
Changes in body composition from baseline to 6 months. BMI, body mass index; LBM, lean body mass.

Mean body weight, BMI, and LBM were all lower at 6 months in patients treated with sorafenib relative to baseline (Fig. 2): mean weight decreased by 5.0 kg (standard deviation [SD] 3.9), mean BMI decreased by 1.8 kg/m^2^ (SD 1.4), and mean LBM decreased by 3.0 kg (SD 2.5). There were no substantial changes in these variables in the placebo group: mean weight decreased by 0.1 kg (SD 3.2), mean BMI was unchanged (SD 1.2), and mean LBM decreased by 0.1 kg (SD 2.2). For all three measures, the decreases were significantly greater in patients receiving sorafenib than in those receiving placebo (*p* < 0.0001).

### Sorafenib toxicity according to sarcopenia status

**FIG. 3.**
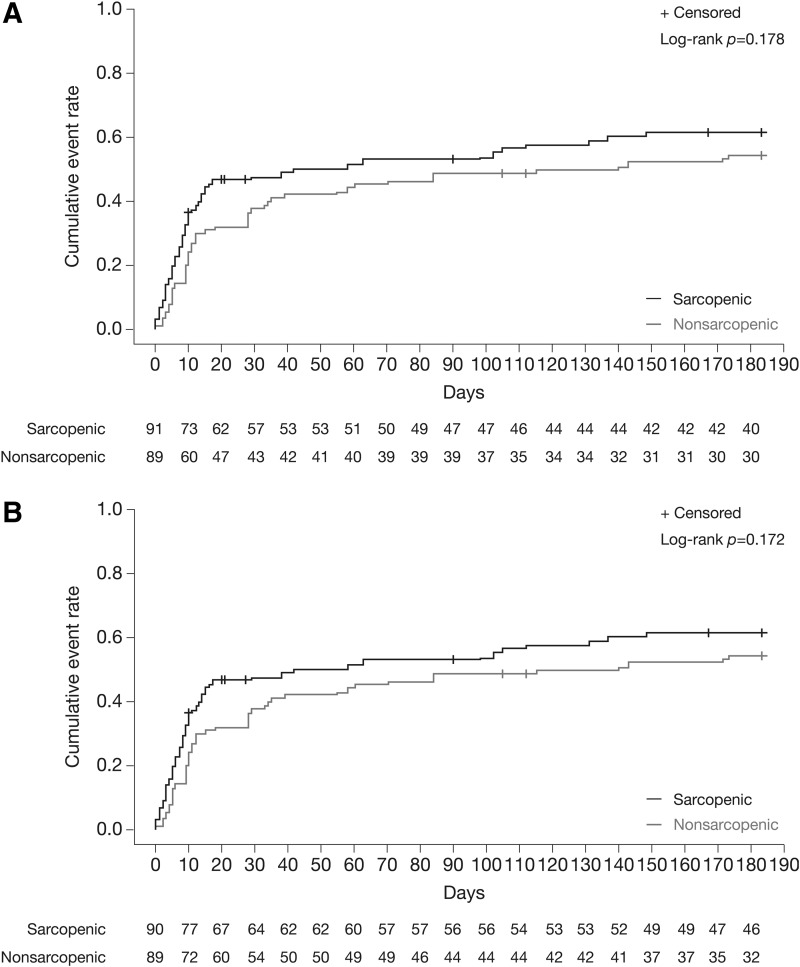
Cumulative rate (Kaplan–Meier) of toxicity leading to dose modification (**A**) and severe toxic events (**B**) in patients treated with sorafenib with and without sarcopenia.

A DMT occurred in 102 patients treated with sorafenib (56.7%). The median time to DMT was 58 days (95% confidence interval [CI]: 13–148) in those with sarcopenia and 140 days in those without sarcopenia (95% CI: 34–NA). The median time to STE was 147 days (95% CI: 58–NA) in those with sarcopenia and was not reached in those without. There was a nonsignificant trend toward a shorter time to DMTs and STEs in sarcopenic patients compared with nonsarcopenic patients (DMT analysis, *p* = 0.178; STE analysis, *p* = 0.172) (Fig. 3). An STE was experienced by 48 patients with sarcopenia (53.9%) compared with 41 without sarcopenia (45.6%); the corresponding figures for DMTs were 53 (59.6%) and 49 (53.8%). Early DMT occurred in 42 patients with sarcopenia (55%) and 34 without sarcopenia (45%). Similar results were observed using definitions A and B for sarcopenia, with no significant correlation observed between sarcopenia and DMTs or STEs (Supplementary Figs. S1 and S2).

Most DMTs and STEs occurred in the first month of treatment (Fig. 3). An early DMT was experienced by 76 of 180 patients treated with sorafenib (42.2%): 42 (55.3%) in patients with sarcopenia and 34 (44.7%) in patients without sarcopenia (*p* = 0.227).

## Discussion

We evaluated whether patients with DTC who are sarcopenic have a higher risk of DMTs during the first month of sorafenib treatment than those without sarcopenia, but found no significant association between sarcopenia and toxicity. Our initial hypothesis was that patients with DTC are more likely to be sarcopenic than patients with other cancers due to long-term thyroxine suppressive therapy. The frequency of sarcopenia in this trial varied widely depending on which definition was used: 54.5% of patients were sarcopenic at baseline using definition A, 30.7% using definition B, and 49.9% using definition C. In comparison, sarcopenia (defined as L3 SM index <52.4 cm^2^/m^2^ for men and <38.5 cm^2^/m^2^ for women) was present in 52.5% of patients with RCC treated with sorafenib (10). Using slightly different definitions of sarcopenia (L3 SM index ≤53 cm^2^/m^2^ for men with a BMI ≥25; ≤43 cm^2^/m^2^ for men with a BMI <25; and ≤41 cm^2^/m^2^ for women), sarcopenia was present in 49% of sorafenib-treated patients with advanced HCC (23).

Patients with sarcopenia receiving sorafenib have an important risk of toxicity that is not significantly different from that of patients without sarcopenia; no associations were found between sarcopenia and DMT or early DMT in these patients with DTC. This contrasts with observations in patients with HCC and RCC, which showed that sarcopenia was associated with toxicity, which might be attributed to differences in underlying disease and prior treatment. For example, patients with RCC may have received interferon, a proinflammatory cytokine that has complex physiologic effects, including effects on SM homeostasis and repair. The link between sarcopenia and toxicity in RCC may be a class effect of multikinase inhibitors because BMI <25 kg/m^2^ and sarcopenia were also predictive of early DMTs with sunitinib in patients with RCC (12); sarcopenia was also predictive of DMT with sunitinib in another RCC study (24). Muscle depletion is a common feature of liver cirrhosis, is present in most patients with HCC, and is an independent prognostic factor.

Body composition may change over time during treatment. In this study, changes were observed over the first 6 months of sorafenib treatment; mean weight, BMI, and LBM were all significantly lower at 6 months in patients in the sorafenib group compared with the placebo group (*p* < 0.0001 for each comparison). Sorafenib significantly improves PFS compared with placebo in patients with progressive radioactive iodine-refractory DTC (16), but its effects on muscle mass, and the associated adverse effects on prognosis, may partially negate these benefits. Consequently, every effort should be made to prevent muscle wasting through nutritional support and appropriate exercise programs (25–27). Conversely, other targeted agents may be associated with gain in muscle mass over time. In a study of patients with medullary thyroid carcinoma, those treated with vandetanib (a VEGFR2 and RET inhibitor) gained body weight and muscle mass at 3 months compared with the placebo group; nevertheless, those with low muscle mass had a higher probability of DMTs (28). Muscle gain was also observed in patients with cholangiocarcinoma treated with the MEK inhibitor selumetinib (29).

The appropriate threshold for defining sarcopenia may vary between different populations defined by age, tumor type, or ethnicity. The widely used definitions are based on data from North American or European populations and may not be adapted to other populations. In the DECISION trial, the incidence of sarcopenia was much higher in Asian patients than in North American or European patients, regardless of which definition was used. Definition C was used in these analyses to include the maximum number of sarcopenic patients who were potentially at a higher risk of toxicity; this definition has not been formally validated, but has the advantage of taking the heterogeneity of the population into account. However, the use of definition C may have contributed to the lack of correlation between sarcopenia (as defined here) and DMT or early DMT.

This study has several limitations, including that it was a retrospective exploratory analysis, not prespecified in the original protocol; analyzable CT scans were not available for all patients, which could have led to bias; and CT scans do not assess function and therefore could be considered inadequate to define sarcopenia. Other limitations include applying the same definition of sarcopenia across all ethnic groups, despite most previous publications being based on Caucasian populations, and that definition C, which was based on the median SM index in this population, has not been formally validated.

In conclusion, while a significant association was observed between sorafenib and reduced muscle mass, no link was seen between sarcopenia and DMT or early DMT in patients with DTC treated with sorafenib in the DECISION trial. Given the growing importance of body composition in oncology, the important variability in body composition in patients with cancer, and the relevance of SM mass for drug dosing (30), further research on the significance of sarcopenia for tyrosine kinase inhibitor toxicity may be warranted.

## Supplementary Material

Supplemental data

Supplemental data

Supplemental data

## Data Sharing Statement

Availability of the data underlying this publication will be determined according to Bayer's commitment to the EFPIA/PhRMA “Principles for responsible clinical trial data sharing”. This pertains to scope, time point, and process of data access. As such, Bayer commits to sharing upon request from qualified scientific and medical researchers patient-level clinical trial data, study-level clinical trial data, and protocols from clinical trials in patients for medicines and indications approved in the United States and European Union as necessary for conducting legitimate research on request from qualified scientific and medical researchers. This applies to data on new medicines and indications approved by the European Union and United States regulatory agencies on or after January 1, 2014. Interested researchers can go to the clinical study data request website to request access to anonymized patient-level data and supporting documents from clinical studies to conduct further research that can help advance medical science or improve patient care. Information on Bayer's criteria for listing studies and other relevant information are provided in the “Study sponsors section” of the portal. Data access will be granted to anonymized patient-level data, protocols, and clinical study reports after approval by an independent scientific review panel. Bayer is not involved in the decisions made by the independent review panel. Bayer will take all necessary measures to ensure that patient privacy is safeguarded.
