# Supplementary material for: Body Composition in Patients with Radioactive Iodine-Refractory, Advanced Differentiated Thyroid Cancer Treated with Sorafenib or Placebo: A Retrospective Analysis of the Phase III DECISION Trial
Source: Thyroid. 2019 Dec 16;29(12):1820–7. doi: 10.1089/thy.2018.0784 (PMC6918875; doi:10.1089/thy.2018.0784)
Supplement: Supplemental data [file Supp_Table1.pdf]

Supplementary Data

Supplementary Table S1. Baseline Characteristics and Body Composition According to Sarcopenia Status

	Sarcopenic (definition A)		*Nonsarcopenic (definition A)*		*Sarcopenic (definition B)*		*Nonsarcopenic (definition B)*		*Sarcopenic (definition C)*		*Nonsarcopenic (definition C)*	
	Placebo (n = *99*)	*Sorafenib* (n = *100*)	*Placebo* (n = *86*)	*Sorafenib* (n = *80*)	*Placebo* (n = *58*)	*Sorafenib* (n = *54*)	*Placebo* (n = *127*)	*Sorafenib* (n = *126*)	*Placebo* (n = *93*)	*Sorafenib* (n = *89*)	*Placebo* (n = *92*)	*Sorafenib* (n = *91*)
Age, years												
Median (range)	65 (33–87)	65 (24–82)	57 (30–80)	62 (27–80)	65 (33–84)	65 (24–82)	61 (30–87)	63 (27–81)	65 (33–87)	65 (24–82)	59 (30–80)	62 (27–80)
Mean (SD)	63.9 (12.0)	63.1 (11.6)	58.0 (10.8)	59.5 (10.8)	62.4 (12.7)	62.4 (12.2)	60.6 (11.4)	61.1 (11.0)	63.5 (12.2)	63.0 (11.5)	58.7 (11.0)	60.1 (11.1)
ECOG PS, *n* (%)												
0	63 (63.6)	59 (59.0)	53 (61.6)	54 (67.5)	35 (60.3)	32 (59.3)	81 (63.8)	81 (64.3)	58 (62.4)	50 (56.2)	58 (63.0)	63 (69.2)
1	33 (33.3)	35 (35.0)	29 (33.7)	25 (31.3)	20 (34.5)	20 (37.0)	42 (33.1)	40 (31.7)	32 (34.4)	33 (37.1)	30 (32.6)	27 (29.7)
2	3 (3.0)	6 (6.0)	3 (3.5)	0	3 (5.2)	2 (3.7)	3 (2.4)	4 (3.2)	3 (3.2)	6 (6.7)	3 (3.3)	0
Missing	0	0	1 (1.2)	1 (1.3)	0	0	1 (0.8)	1 (0.8)	0	0	1 (1.1)	1 (1.1)
Time since diagnosis, months												
Median (range)	83 (9–402)	61 (4–363)	60 (7–288)	72 (4–348)	83 (11–363)	67 (4–339)	64 (7–402)	67 (4–363)	97 (13–402)	66 (4–363)	54 (7–325)	67 (4–348)
Mean (SD)	103.3 (77.5)	81.7 (76.0)	81.7 (59.9)	86.3 (69.2)	102.9 (70.9)	82.9 (73.1)	89.0 (70.2)	84.2 (73.0)	109.6 (73.7)	86.5 (75.0)	76.7 (63.3)	81.0 (71.0)
Weight, kg												
Median (range)	68 (44–125)	71 (35–120)	79 (42–142)	78 (44–140)	63 (44–81)	60 (35–81)	82 (41–142)	82 (44–140)	66 (44–125)	66 (35–120)	82 (42–142)	83 (44–140)
Mean (SD)	70.5 (15.8)	72.0 (17.8)	81.4 (21.1)	80.2 (17.6)	61.3 (9.3)	59.3 (10.0)	82.1 (19.1)	82.7 (16.3)	68.5 (14.8)	68.5 (15.9)	82.7 (20.6)	82.6 (17.6)
BMI, kg/m^2												
Median (range)	24 (16–39)	24 (16–35)	29 (17–48)	29 (18–45)	22 (16–25)	22 (16–25)	28 (17–48)	28 (18–45)	24 (16–39)	23 (16–35)	29 (17–48)	29 (18–45)
Mean (SD)	24.7 (4.0)	24.7 (4.1)	29.9 (6.5)	29.3 (5.0)	22.1 (2.0)	21.5 (2.0)	29.4 (5.6)	29.0 (4.3)	24.4 (4.0)	24.1 (3.8)	29.9 (6.2)	29.3 (4.9)
BMI group, *n* (%)												
<18.5 kg/m^2	3 (3.0)	6 (6.0)	2 (2.3)	1 (1.3)	3 (5.2)	6 (11.1)	2 (1.6)	1 (0.8)	3 (3.2)	6 (6.7)	2 (2.2)	1 (1.1)
18.5–<25 kg/m^2	55 (55.6)	48 (48.0)	18 (20.9)	16 (20.0)	55 (94.8)	48 (88.9)	18 (14.2)	16 (12.7)	56 (60.2)	48 (53.9)	17 (18.5)	16 (17.6)
25 to <30 kg/m^2	28 (28.3)	36 (36.0)	30 (34.9)	30 (37.5)	0	0	58 (45.7)	66 (52.4)	24 (25.8)	31 (34.8)	34 (37.0)	35 (38.5)
≥30 kg/m^2	13 (13.1)	10 (10.0)	36 (41.9)	33 (41.3)	0	0	49 (38.6)	43 (34.1)	10 (10.8)	4 (4.5)	39 (42.4)	39 (42.9)
SM index, cm^2/m^2												
Median (range)	41 (25–55)	42 (26–55)	47 (39–81)	51 (39–75)	38 (25–53)	38 (26–54)	47 (29–81)	49 (32–75)	39 (25–51)	39 (26–51)	53 (40–81)	53 (40–75)
Mean (SD)	41.6 (7.7)	42.3 (7.8)	50.7 (10.0)	51.3 (8.5)	39.5 (6.9)	38.8 (6.8)	48.7 (9.7)	49.6 (8.2)	39.5 (6.1)	39.9 (6.2)	52.2 (8.9)	52.6 (7.2)
LBM, kg												
Median (range)	43 (25–60)	44 (27–64)	43 (32–79)	46 (34–74)	38 (25–53)	36 (27–55)	46 (29–79)	49 (32–74)	38 (25–58)	38 (27–58)	49 (32–79)	52 (34–74)
Mean (SD)	41.8 (9.2)	43.2 (9.7)	47.6 (11.1)	48.5 (10.9)	39.1 (7.6)	38.2 (7.8)	47.0 (10.7)	48.7 (10.0)	39.6 (7.8)	40.1 (7.9)	49.6 (10.4)	50.9 (10.2)

BMI, body mass index; ECOG PS, Eastern Cooperation Oncology Group performance status; LBM, lean body mass; SD, standard deviation; SM, skeletal muscle.
