# Supplementary material for: Body Composition in Patients with Radioactive Iodine-Refractory, Advanced Differentiated Thyroid Cancer Treated with Sorafenib or Placebo: A Retrospective Analysis of the Phase III DECISION Trial
Source: Thyroid. 2019 Dec 16;29(12):1820–7. doi: 10.1089/thy.2018.0784 (PMC6918875; doi:10.1089/thy.2018.0784)
Supplement: Supplemental data [file Supp_Fig1.pdf]

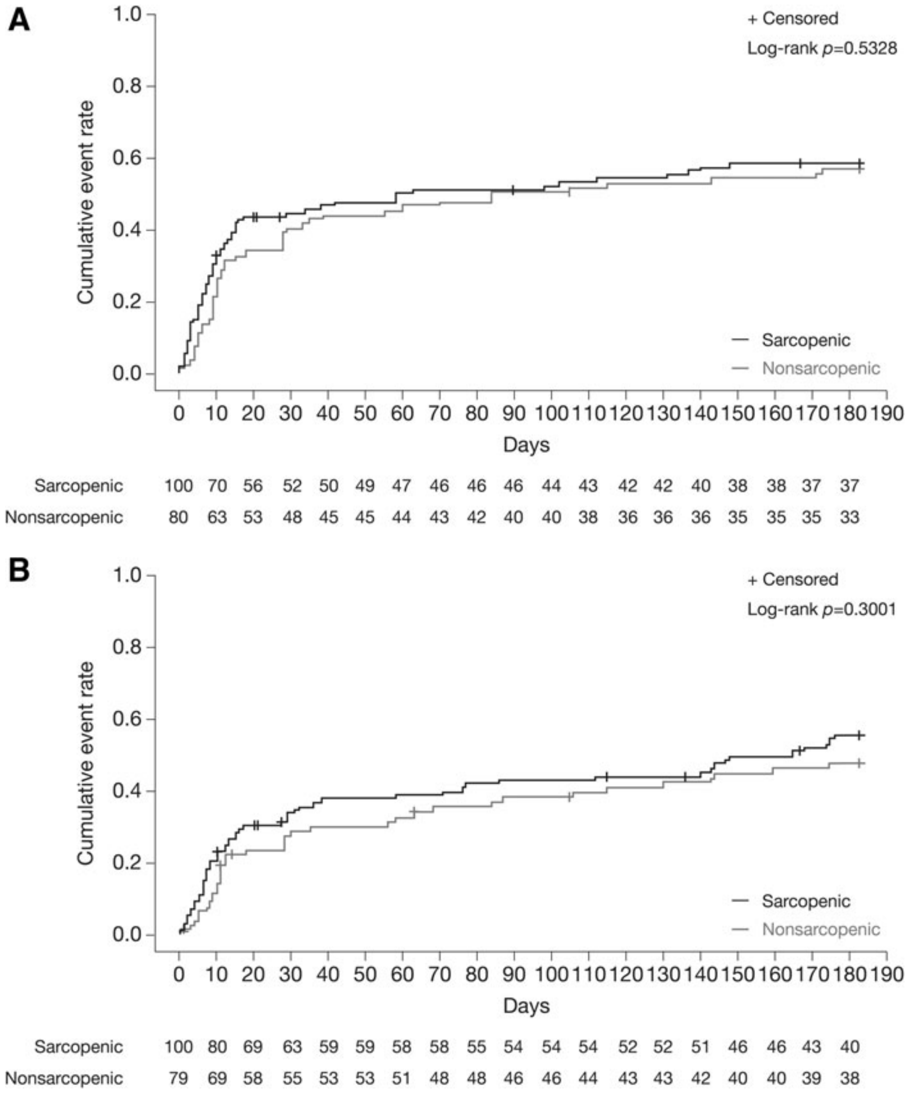

SUPPLEMENTARY FIG. S1. Cumulative rate (Kaplan–Meier) of toxicity leading to dose modifications (**A**) and severe toxic events (**B**) in sorafenib-treated patients with and without sarcopenia (definition A).
